# P-2135. Pneumocystis jirovecii Pneumonia in Hematologic Malignancies: A 15-Year Retrospective Cohort

**DOI:** 10.1093/ofid/ofaf695.2299

**Published:** 2026-01-11

**Authors:** Cristina Ruiz, Isabel Ramirez-Sanchez

**Affiliations:** Hospital Pablo Tobon Uribe, Medellin, Antioquia, Colombia; Universidad de Antioquia, Hospital Pablo Tobon Uribe, Medellin, Antioquia, Colombia

## Abstract

**Background:**

*Pneumocystis jirovecii* pneumonia (PJP) is an opportunistic fungal pneumonia that affects immunosuppressed individuals. The increasing use of immunosuppressive therapies for the treatment of hematologic malignancies (HM) and graft-versus-host disease (GVHD) in hematopoietic stem cell transplantation (HSCT) has led to an increase in the incidence of PJP. In this population, the sensitivity of traditional histologic and microscopic diagnostic tests is lower.

**Figure:**
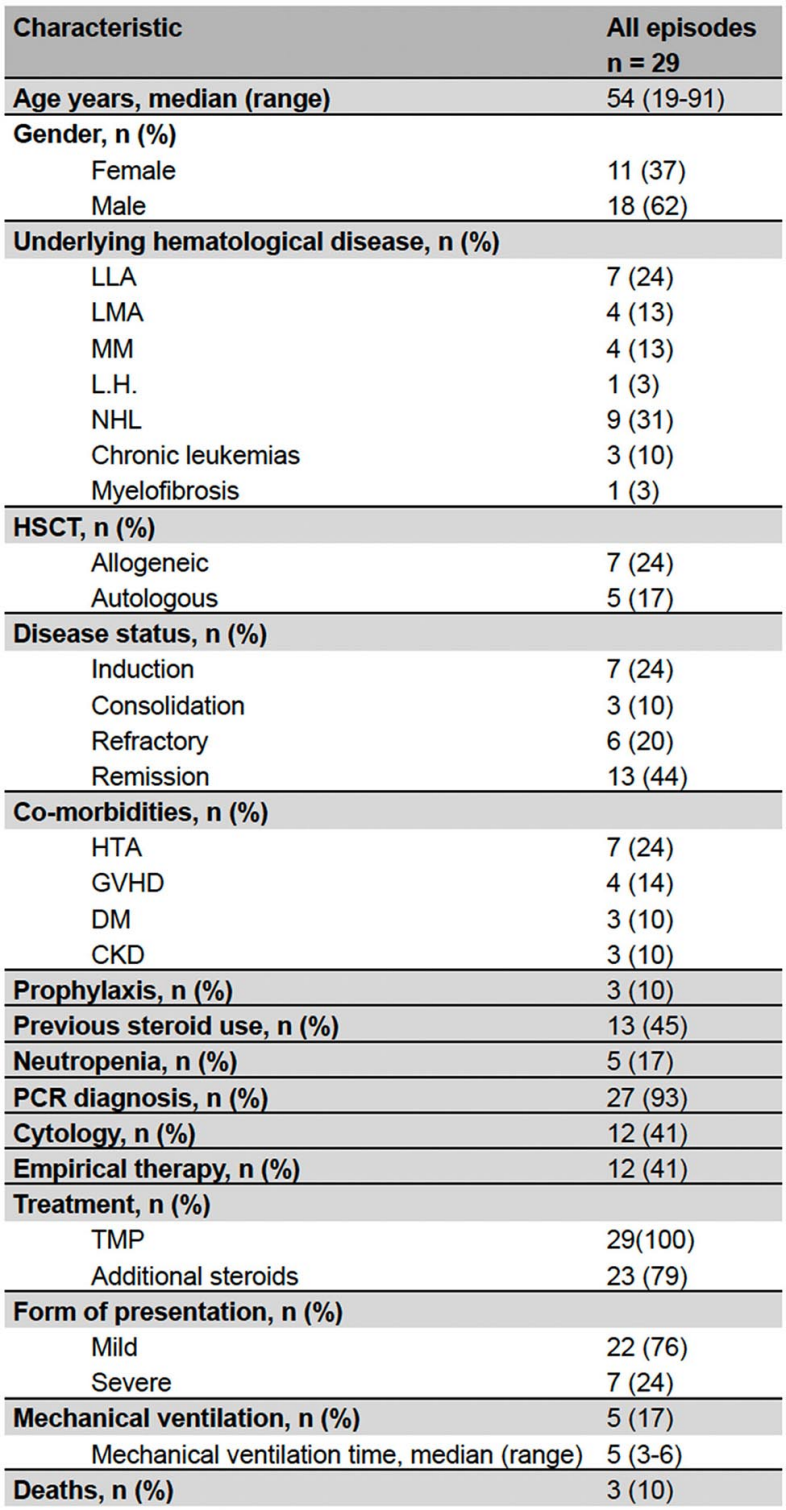
Demographic characteristics of PCP patients in hematologic malignancies

**Methods:**

To describe the characteristics of patients with HM who underwent diagnostic testing for PJP and to evaluate demographic and clinical variables, assess the performance of PCR and cytology for *Pneumocystis jirovecii* in respiratory specimens, and describe 30-day mortality in a high-level complex hospital in Medellin Colombia. PJP was defined as the detection of *Pneumocystis jirovecii* according to the EORTC/MSGERC definitions.

**Results:**

10,307 patients were treated in the hematology department, 29 of whom had possible or confirmed PJP, with a cumulative incidence of 0.28%. Ninety-six percent were classified as confirmed disease. The most common underlying HM was non-Hodgkin lymphoma (31%). 41.3% of cases were HSCT recipients, 58% of whom were allogeneic. Fifty-seven percent of them had a history of GVHD. Forty-six percent of patients were exposed to steroids at the time of infection, and only 18% presented neutropenia. Fifteen of those who received steroids were not receiving prophylaxis.

The clinical presentation was mild in 75% of cases. All patients underwent fiberoptic bronchoscopy, ninety-six percent of cases were confirmed by PCR in bronchoalveolar lavage (BAL), and cytology was positive in only 43%. All patients received treatment with trimethoprim-sulfamethoxazole and 79% received concomitant steroid treatment. Mortality was 10%.

**Conclusion:**

PJP in HM has a low incidence, and the mortality rate in our population is low compared to other cohorts. It is not associated with neutropenia. In most cases, the presentation was mild, and in HSCT, it manifests in late stages. Since most cases did not receive prophylaxis, each chemotherapy regimen or comorbidity must be individualized to determine its appropriateness.

**Disclosures:**

All Authors: No reported disclosures

